# Supplementary material for: Higher body condition with infection by Haemoproteus parasites in Bananaquits (Coereba flaveola)
Source: PeerJ. 2024 Mar 29;12:e16361. doi: 10.7717/peerj.16361 (PMC10984167; doi:10.7717/peerj.16361)
Supplement: Supplemental Information 3 [file peerj-12-16361-s003.zip › Sequences_ConsequencesInfection_Bananaquit/metadata/Table_Data_ConsquencesInfection_Bana.docx]

**Table 1.** Data and sequence name of infected Bananaquits.

| **ID** | **ALPHA** | **Sex** | **Age** | **Infection status** | **Name of sequence file** |
| --- | --- | --- | --- | --- | --- |
| 034 | BANA | U | HY | Infected |  |
| 038 | BANA | M | AHY | Infected |  |
| 044 | BANA | M | AHY | Infected |  |
| 059 | BANA | M | AHY | Infected |  |
| 073 | BANA | M | AHY | Infected |  |
| 082 | BANA | M | AHY | Infected |  |
| 121 | BANA | M | AHY | Infected |  |
| 131 | BANA | U | HY | Infected |  |
| 132 | BANA | M | AHY | Infected |  |
| 133 | BANA | U | AHY | Infected |  |
| 162 | BANA | M | AHY | Infected |  |
| 169 | BANA | U | AHY | Infected |  |
| 174 | BANA | U | HY | Infected |  |
| 197 | BANA | U | AHY | Infected |  |
| 198 | BANA | F | AHY | Infected |  |
