# Supplementary material for: Higher body condition with infection by Haemoproteus parasites in Bananaquits (Coereba flaveola)
Source: PeerJ. 2024 Mar 29;12:e16361. doi: 10.7717/peerj.16361 (PMC10984167; doi:10.7717/peerj.16361)
Supplement: Supplemental Information 4 [file peerj-12-16361-s004.docx]

**SUPPLEMENTAL MATERIAL**


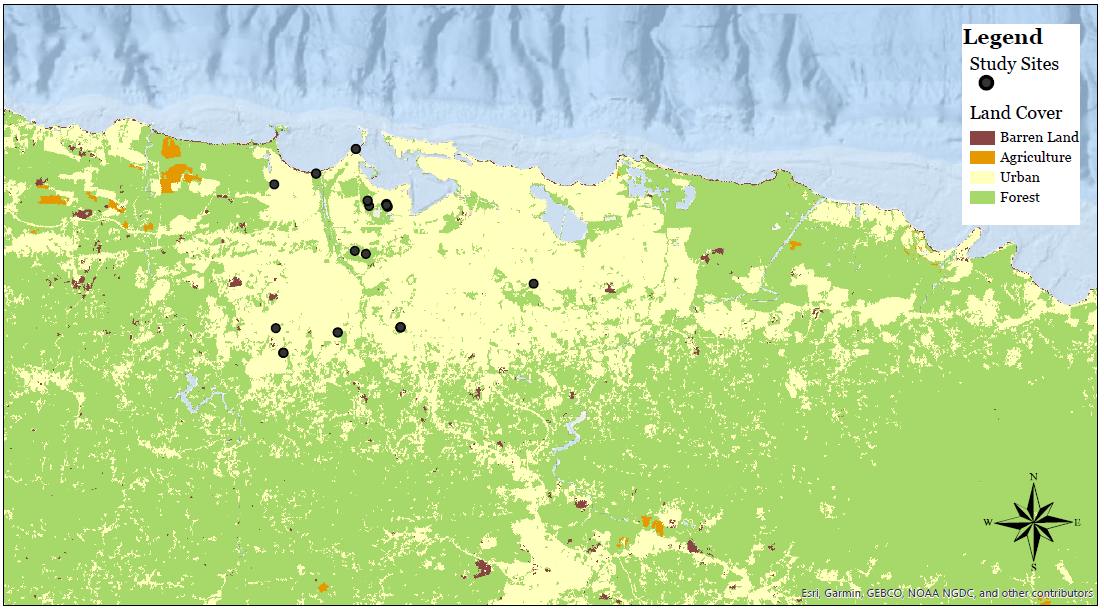


**Figure S1.** The map shows the study sites distribution in the urban land cover areas of Puerto Rico. Study sites (n=13) are marked with a dark grey dot.


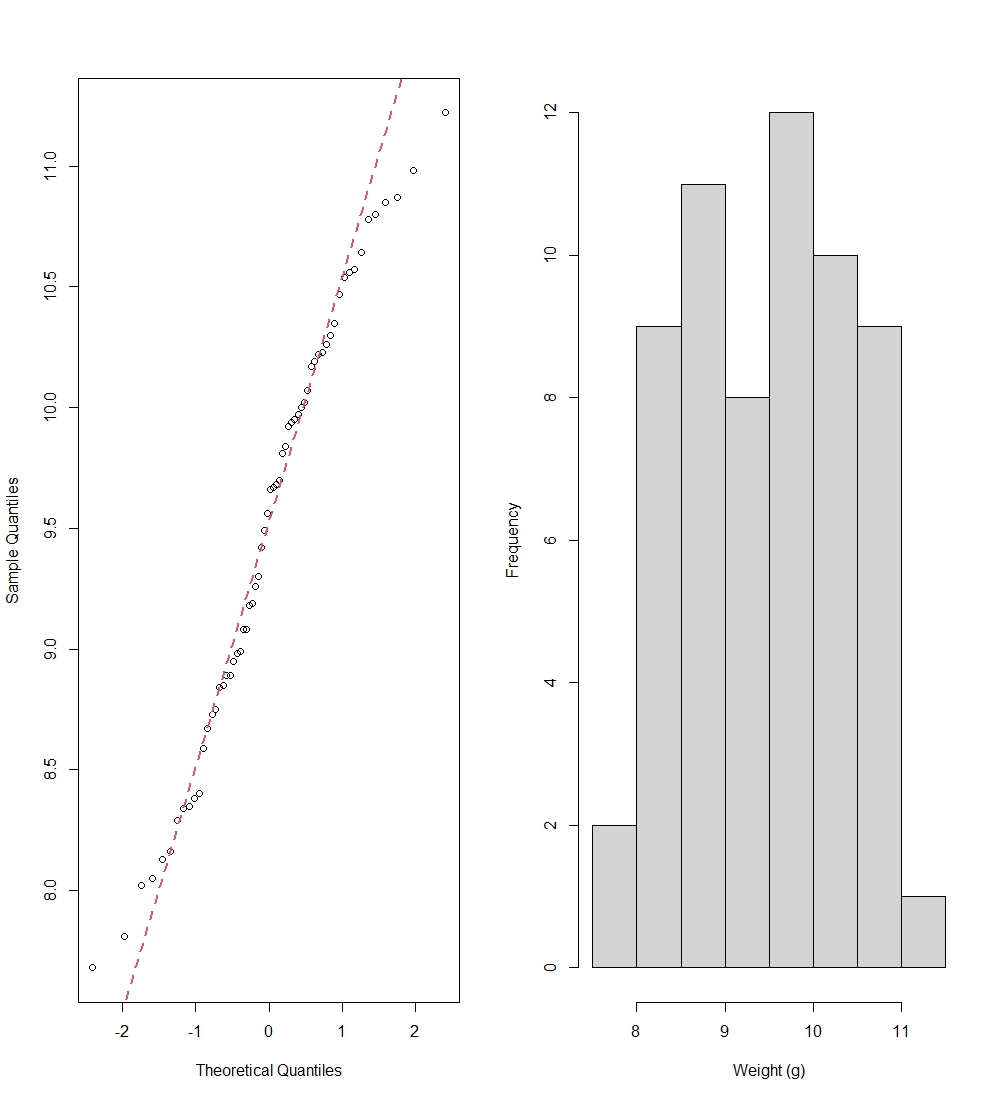


**Figure S2**. Distribution of body weights.


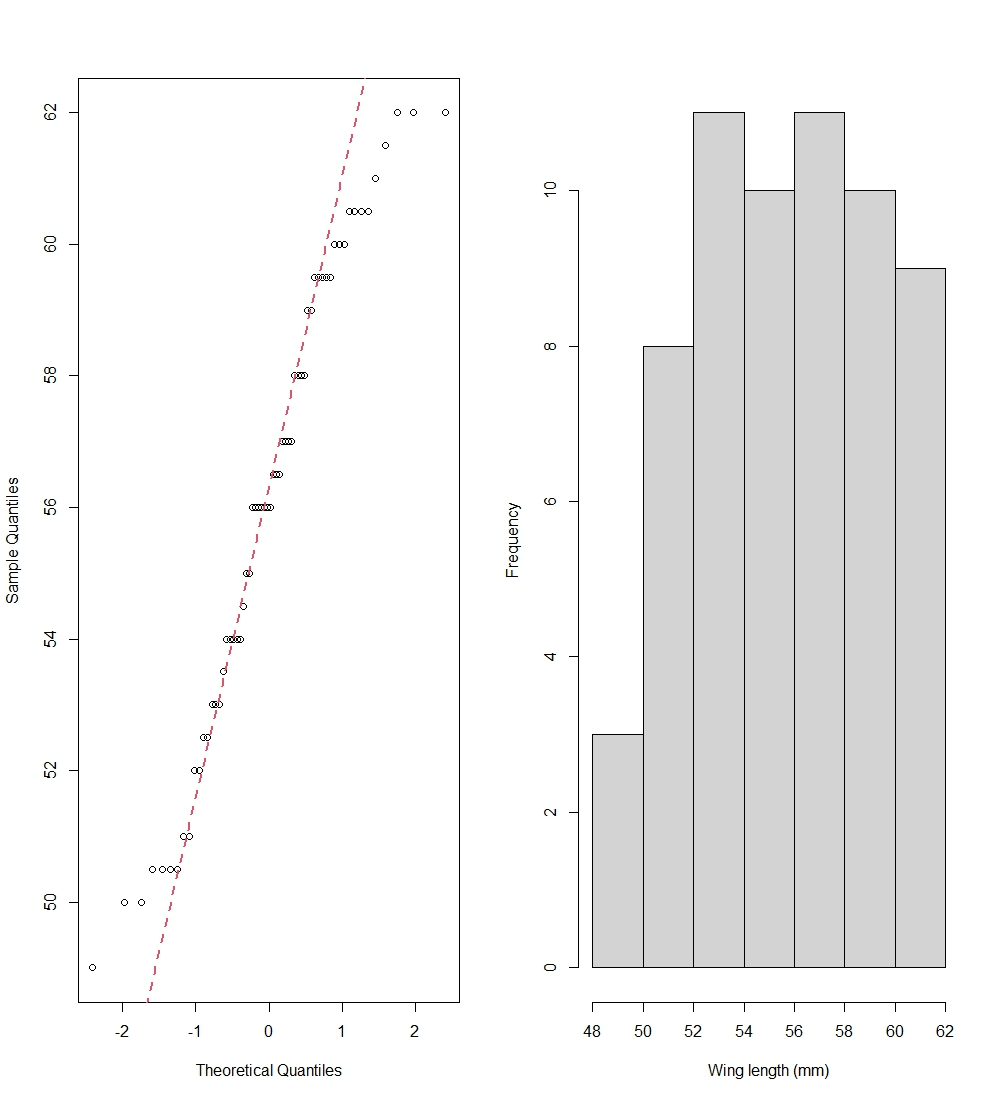


**Figure S3**. Distribution of wing lengths


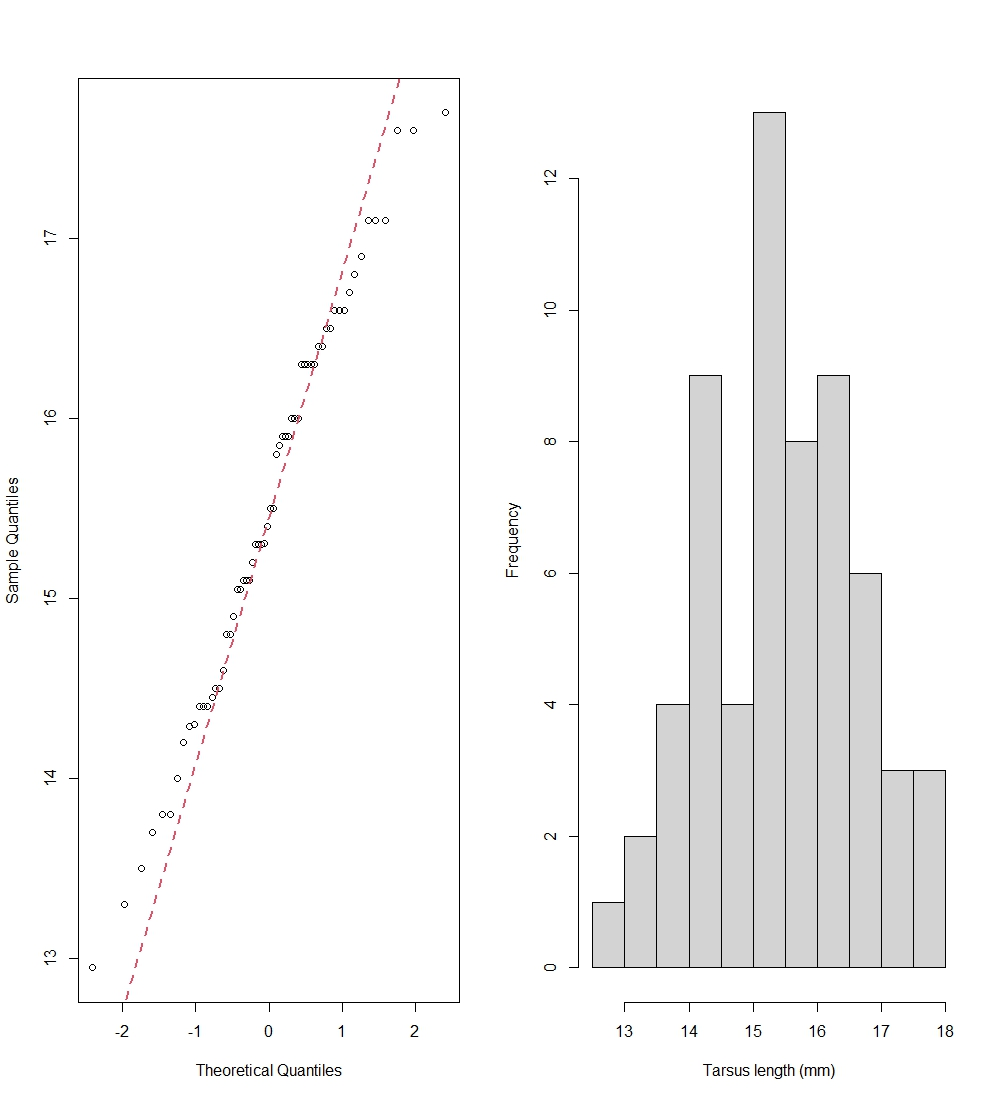


**Figure S4**. Distribution of tarsus lengths


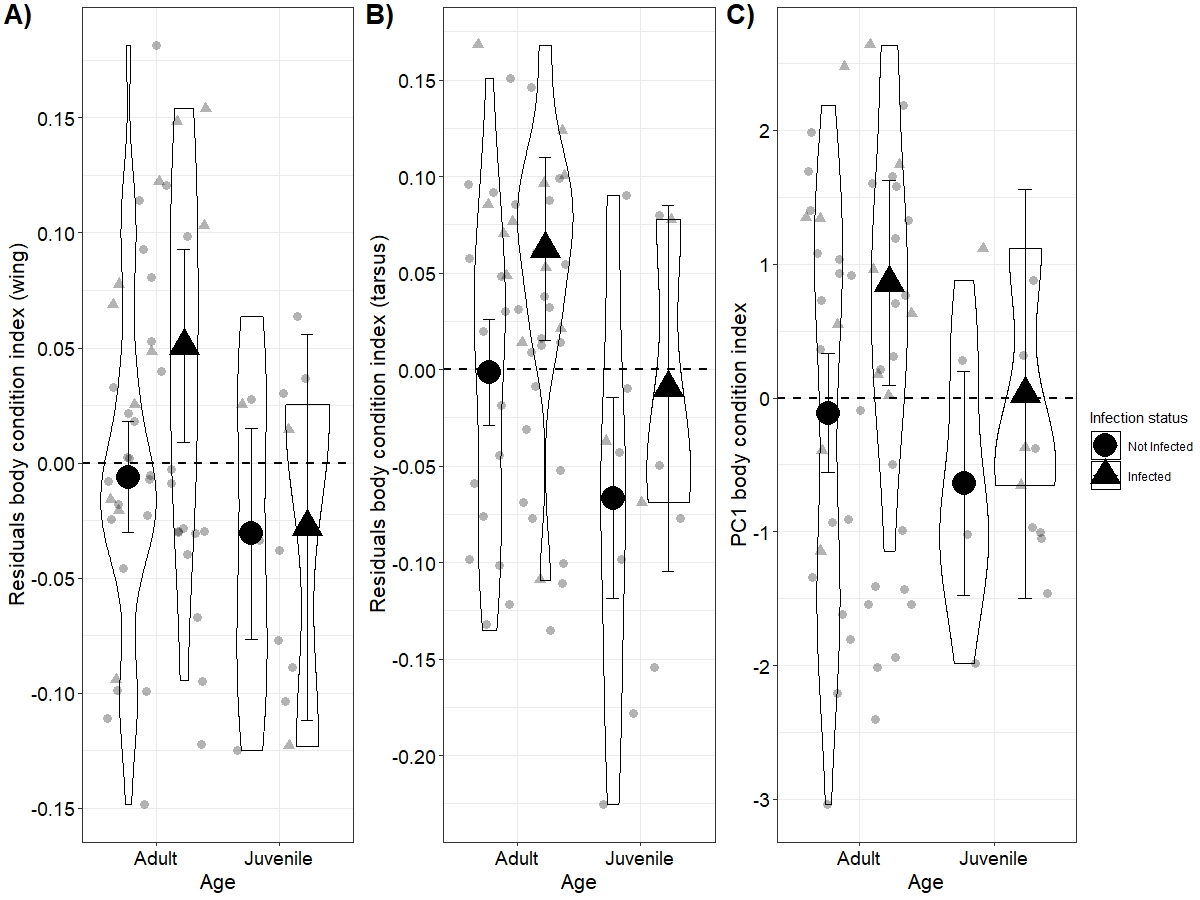


**Figure S5.** Comparison of body condition of infected and uninfected juvenile (HY; n=13) and adult (AHY; n=47) Bananaquits using a A) residual body condition index of wing B) residual body condition index of tarsus and C) PCA body condition index. The horizontal dash at zero represents average body condition. Individuals above the line have higher body mass than average, while individuals below the line have lower body mass than average. Jittered dots indicate individuals included in the analysis for the respective classification of non-infected and infected adults and juveniles. The lines inside violin plots indicate the 95% confidence intervals, dot indicates the point estimate of the model of the non-infected individuals and triangle indicates the point estimate of the model of the infected individuals.


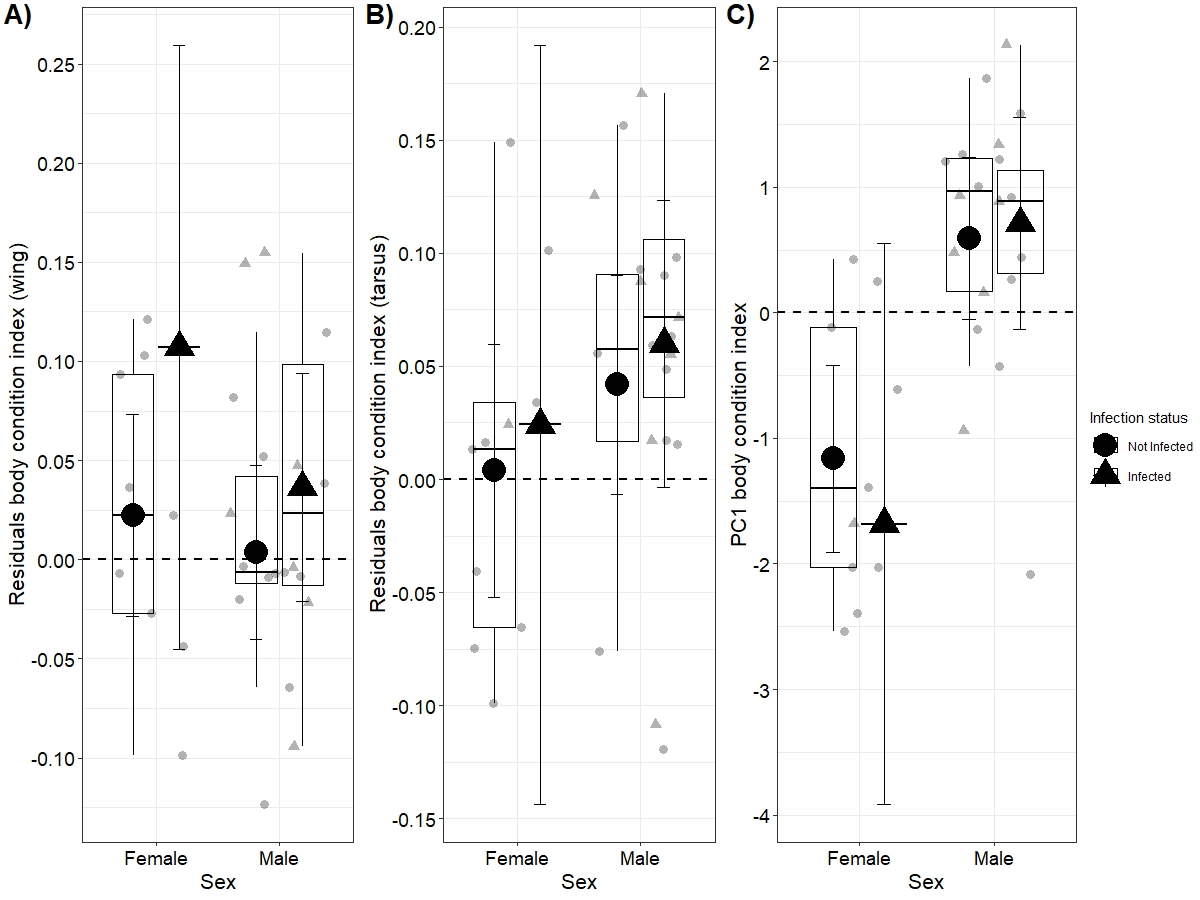


**Figure S6.** Comparison of body condition of infected and uninfected male (M; n=19) and female (F; n=10) Bananaquits using a A) residual body condition index of wing B) residual body condition index of tarsus and C) PCA body condition index. The horizontal dash at zero represents average body condition. Individuals above the line have higher body mass than average, while individuals below the line have lower body mass than average. Jittered dots indicate individuals included in the analysis for the respective classification of non-infected and infected males (M) and females (F). The lines inside the violin plots indicate the 95% confidence intervals, dot indicates the point estimate of the model of the non-infected individuals and triangle indicates the point estimate of the model of the infected individuals.

**Table S1.** Project study sites (n=13) with the municipality (n=5) where they are located, coordinates and number of trials (n=26) in each site.

| **Site Name** | **Municipality** | **Dates sampled** | **Coordinates** | **Number of trials** |
| --- | --- | --- | --- | --- |
| Interamerican University | Bayamón | 30-June-2018  31-July-2018  22-August-2018 | Lat: 18°21'17.45"N, Lon: 66°10'56.26"W | 3 |
| Rexville | Bayamón | 19-October-2018 | Lat: 18°22'4.99"N, Lon: 66°11'9.06"W | 1 |
| Julio Enrique Monagas Park | Bayamón | 19-December-2018  4-January-2019 | Lat: 18°24'30.76"N, Lon: 66° 8'41.80"W | 2 |
| Near ASSMCA | Bayamón | 21-September-2018  24-October-2018 | Lat: 18°21'56.61"N, Lon: 66° 9'3.86"W | 2 |
| Near Kimberly Clark | Cataño | 26-July-2018  7-September-2018 | Lat: 18°25'57.73"N, Lon: 66° 7'24.34"W | 2 |
| Community Garden | Cataño | 12-October-2018 | Lat: 18°25'59.63"N, Lon: 66° 8'1.11"W | 1 |
| Laguna Secreta | Cataño | 30-November-2018  10-December-2018  12-December-2018 | Lat: 18°26'1.32"N, Lon: 66° 7'26.80"W | 3 |
| Corredor Yaguazo | Cataño | 21-December-2018  5-January-2019 | Lat: 18°26'9.67"N, Lon: 66° 8'4.44"W | 2 |
| Near Humane Society | Guaynabo | 8-September-2018  22-September-2018  6-October-2018  15-December-2018 | Lat: 18°22'4.84"N, Lon: 66° 6'59.46"W | 4 |
| Reparto Universitario | San Juan | 4-August-2018 | Lat: 18°23'29.75"N, Lon: 66° 2'29.24"W | 1 |
| Isla de Cabras | Toa Baja | 3-July-2018  2-August-2018 | Lat: 18°27'48.63"N, Lon: 66° 8'28.55"W | 2 |
| Levittown Bridge | Toa Baja | 28-July-2018  14-August-2018 | Lat: 18°26'59.37"N, Lon: 66° 9'50.35"W | 2 |
| Levittown 5^th^ section | Toa Baja | 28-July-2018 | Lat: 18°26'40.60"N, Lon: 66°11'13.77"W | 1 |

**Table S2.** Data of individual Bananaquits captured during the study sampling period and used for the analysis. External anatomy characteristics quantified as in the MAPS program manual.

| **ID** | **ALPHA** | **SP** | **CP** | **BP** | **BF** | **Sex** | **Age** |
| --- | --- | --- | --- | --- | --- | --- | --- |
| 015 | BANA | 8 | 0 | 0 | 0 | M | AHY |
| 017 | BANA | 2 | 0 | 5 | 0 | F | AHY |
| 026 | BANA | 3 | 0 | 1 | 0 | U | HY |
| 029 | BANA | 8 | 0 | 5 | 0 | F | AHY |
| 030 | BANA | 6 | 1 | 0 | 0 | U | AHY |
| 033 | BANA | 6 | 2 | 0 | 0 | M | AHY |
| 034 | BANA | 2 | 0 | 0 | 0 | U | HY |
| 036 | BANA | 6 | 0 | 0 | 0 | U | AHY |
| 038 | BANA | 8 | 2 | 0 | 0 | M | AHY |
| 040 | BANA | 5 | 0 | 0 | 0 | U | AHY |
| 041 | BANA | 6 | 0 | 0 | 0 | U | AHY |
| 044 | BANA | 6 | 1 | 0 | 0 | M | AHY |
| 049 | BANA | 3 | 0 | 0 | 0 | U | HY |
| 053 | BANA | 3 | 0 | 0 | 0 | U | HY |
| 054 | BANA | 3 | 0 | 0 | 0 | U | HY |
| 059 | BANA | 5 | 1 | 0 | 0 | M | AHY |
| 072 | BANA | 5 | 1 | 0 | 0 | M | AHY |
| 073 | BANA | 6 | 0 | 0 | 0 | U | AHY |
| 076 | BANA | 3 | 0 | 0 | 0 | U | HY |
| 078 | BANA | 5 | 0 | 0 | 0 | U | AHY |
| 079 | BANA | 8 | 0 | 1 | 0 | F | AHY |
| 080 | BANA | 8 | 0 | 0 | 0 | U | U |
| 081 | BANA | 6 | 1 | 0 | 0 | M | AHY |
| 082 | BANA | 6 | 1 | 0 | 0 | M | AHY |
| 094 | BANA | 6 | 0 | 0 | 0 | U | AHY |
| 106 | BANA | 6 | 0 | 0 | 0 | U | AHY |
| 108 | BANA | 6 | 0 | 0 | 0 | M | AHY |
| 112 | BANA | 3 | 0 | 0 | 0 | U | HY |
| 121 | BANA | 6 | 1 | 0 | 0 | M | AHY |
| 122 | BANA | 4 | 0 | 2 | 0 | F | AHY |
| 124 | BANA | 6 | 0 | 0 | 0 | U | AHY |
| 130 | BANA | 1 | 0 | 0 | 0 | U | HY |
| 131 | BANA | 4 | 0 | 0 | 0 | U | HY |
| 132 | BANA | 6 | 2 | 0 | 0 | M | AHY |
| 133 | BANA | 5 | 0 | 0 | 0 | U | AHY |
| 138 | BANA | 5 | 0 | 0 | 0 | U | AHY |
| 139 | BANA | 6 | 2 | 0 | 0 | M | AHY |
| 140 | BANA | 8 | 0 | 0 | 0 | U | AHY |
| 146 | BANA | 6 | 1 | 0 | 1 | M | AHY |
| 151 | BANA | 2 | 0 | 0 | 0 | U | HY |
| 152 | BANA | 4 | 0 | 0 | 0 | U | AHY |
| 156 | BANA | 5 | 0 | 0 | 0 | U | AHY |
| 161 | BANA | 6 | 0 | 2/3 | 0 | F | AHY |
| 162 | BANA | 6 | 1 | 0 | 0 | M | AHY |
| 163 | BANA | 3 | 0 | 0 | 0 | U | HY |
| 164 | BANA | 6 | 0 | 3 | 0 | F | AHY |
| 166 | BANA | 6 | 1 | 0 | 0 | M | AHY |
| 169 | BANA | 6 | 0 | 0 | 2 | U | AHY |
| 170 | BANA | 4 | 0 | 0 | 0 | U | AHY |
| 172 | BANA | 6 | 2 | 0 | 0 | M | AHY |
| 174 | BANA | 3 | 0 | 0 | 0 | U | HY |
| 182 | BANA | 3 | 0 | 5 | 0 | F | AHY |
| 185 | BANA | 4 | 0 | 3 | 0 | F | AHY |
| 192 | BANA | 5 | 3 | 0 | 0 | M | AHY |
| 194 | BANA | 5 | 0 | 0 | 0 | U | AHY |
| 195 | BANA | 5 | 0 | 4 | 0 | F | AHY |
| 196 | BANA | 4 | 1 | 0 | 0 | M | AHY |
| 197 | BANA | 5 | 0 | 0 | 1 | U | AHY |
| 198 | BANA | 5 | 0 | 2 | 1 | F | AHY |
| 199 | BANA | 5 | 1 | 0 | 0 | M | AHY |
| 200 | BANA | 3 | 0 | 0 | 0 | U | HY |
| 202 | BANA | 4/5 | 0 | 0 | 0 | U | AHY |

**Table S3a.** Results of the linear model predicting log (weight) as a function of log (wing length). The residuals from this model are used to estimate body condition.

|  | log(weight) ~ log(wing length) | | |
| --- | --- | --- | --- |
| *Predictors* | *Estimates* | *CI* | *p* |
| (Intercept) | -1.50 | -2.70 – -0.29 | 0.016 |
| wing length [log] | 0.93 | 0.63 – 1.23 | <0.001 |
| Observations | 62 | | |
| R2 / R2 adjusted | 0.390 / 0.380 | | |

**Table S3b**. Results of the linear model predicting log (weight) as a function of log (tarsus length). The residuals from this model are used to estimate body condition.

|  | log(weight) ~ log(tarsus length) | | |
| --- | --- | --- | --- |
| *Predictors* | *Estimates* | *CI* | *p* |
| (Intercept) | 0.97 | 0.11 – 1.82 | 0.028 |
| tarsus length [log] | 0.47 | 0.15 – 0.78 | 0.004 |
| Observations | 62 | | |
| R2 / R2 adjusted | 0.129 / 0.115 | | |

**Table S4a**. Mass-wing body condition index as a function of parasite infection.

|  | BCI-wing ~ Infection | | |
| --- | --- | --- | --- |
| *Predictors* | *Estimates* | *CI* | *p* |
| (Intercept) | -0.01 | -0.03 – 0.01 | 0.293 |
| parasite [Infected] | 0.05 | 0.00 – 0.09 | 0.035 |
| Observations | 62 | | |
| R2 / R2 adjusted | 0.072 / 0.056 | | |

**Table S4b**. Mass-tarsus body condition index as a function of parasite infection.

|  | BCI-tarsus ~ Infection | | |
| --- | --- | --- | --- |
| *Predictors* | *Estimates* | *CI* | *p* |
| (Intercept) | -0.02 | -0.04 – 0.01 | 0.208 |
| parasite [Infected] | 0.07 | 0.02 – 0.12 | 0.012 |
| Observations | 62 | | |
| R2 / R2 adjusted | 0.100 / 0.085 | | |

**Table S4c**. Fixed effects linear model of PCA body condition index predicted by infection status.

|  | BCI-PCA ~ Infection | | |
| --- | --- | --- | --- |
| *Predictors* | *Estimates* | *CI* | *p* |
| (Intercept) | -0.23 | -0.62 – 0.16 | 0.238 |
| parasite [Infected] | 0.96 | 0.17 – 1.75 | 0.018 |
| Observations | 62 | | |
| R2 / R2 adjusted | 0.089 / 0.074 | | |

**Table S5a**. Fixed effects of mass and wing length residuals linear model predicted by infection status interacting with age.

| BCI-wing ~ Infection*Age | | |  |
| --- | --- | --- | --- |
| *Predictors* | *Estimates* | *CI* | *p* |
| (Intercept) | -0.01 | -0.03 – 0.02 | 0.614 |
| parasite [Infected] | 0.06 | 0.01 – 0.11 | 0.022 |
| age [HY] | -0.02 | -0.08 – 0.03 | 0.346 |
| parasite [Infected] × age [HY] | -0.05 | -0.16 – 0.05 | 0.316 |
| Observations | 61 | | |
| R2 / R2 adjusted | 0.129 / 0.083 | | |

**Table S5b.** Fixed effects linear model of mass and tarsus length residuals predicted by infection status interacting with age.

| BCI-tarsus ~ Infection*Age | | |  |
| --- | --- | --- | --- |
| *Predictors* | *Estimates* | *CI* | *p* |
| (Intercept) | -0.00 | -0.03 – 0.03 | 0.912 |
| parasite [Infected] | 0.06 | 0.01 – 0.12 | 0.023 |
| age [HY] | -0.07 | -0.12 – -0.01 | **0.030** |
| parasite [Infected] × age [HY] | -0.01 | -0.13 – 0.11 | 0.910 |
| Observations | 61 | | |
| R2 / R2 adjusted | 0.193 / 0.150 | | |

**Table S5c.** Fixed effects linear model of PCA body condition index predicted by infection status interacting with age.

|  | BCI-PCA ~ Infection * Age | | |
| --- | --- | --- | --- |
| *Predictors* | *Estimates* | *CI* | *p* |
| (Intercept) | -0.11 | -0.55 – 0.33 | 0.613 |
| parasite [Infected] | 0.97 | 0.09 – 1.86 | 0.032 |
| age [HY] | -0.53 | -1.47 – 0.42 | 0.271 |
| parasite [Infected] × age [HY] | -0.30 | -2.26 – 1.65 | 0.756 |
| Observations | 61 | | |
| R2 / R2 adjusted | 0.118 / 0.072 | | |

**Table S6a.** Fixed effects model of mass and wing length residuals predicted by infection status interacting with sex.

|  | **BCI-wing ~ Infection * Sex** | | |
| --- | --- | --- | --- |
| *Predictors* | *Estimates* | *CI* | *p* |
| (Intercept) | 0.02 | -0.03 – 0.07 | 0.376 |
| parasite [Infected] | 0.08 | -0.08 – 0.25 | 0.287 |
| sex [M] | -0.02 | -0.09 – 0.05 | 0.577 |
| parasite [Infected] × sex [M] | -0.05 | -0.23 – 0.12 | 0.547 |
| Observations | 29 | | |
| R^2^ / R^2^ adjusted | 0.084 / -0.025 | | |

**Table S6b**. Fixed effects model of mass and tarsus length residuals predicted by infection status interacting with sex.

|  | **BCI-tarsus ~ Infection * Sex** | | |
| --- | --- | --- | --- |
| *Predictors* | *Estimates* | *CI* | *p* |
| (Intercept) | 0.00 | -0.05 – 0.06 | 0.889 |
| parasite [Infected] | 0.02 | -0.16 – 0.20 | 0.815 |
| sex [M] | 0.04 | -0.04 – 0.11 | 0.301 |
| parasite [Infected] × sex [M] | -0.00 | -0.20 – 0.19 | 0.981 |
| Observations | 29 | | |
| R^2^ / R^2^ adjusted | 0.076 / -0.035 | | |

**Table S6c.** Fixed effects linear model of PCA body condition index predicted by infection status interacting with sex.

|  | **BCI-PCA ~ Infection * Sex** | | |
| --- | --- | --- | --- |
| *Predictors* | *Estimates* | *CI* | *p* |
| (Intercept) | -1.16 | -1.90 – -0.41 | **0.004** |
| parasite [Infected] | -0.52 | -2.88 – 1.83 | 0.652 |
| sex [M] | 1.76 | 0.77 – 2.74 | **0.001** |
| parasite [Infected] × sex [M] | 0.64 | -1.95 – 3.22 | 0.615 |
| Observations | 29 | | |
| R^2^ / R^2^ adjusted | 0.436 / 0.368 | | |

**SI Article.** Molecular diagnostics detailed protocol

We used coral load for the initial diagnostics of the samples because it catalyzes the reaction and aids in the detection of samples with low parasite load. After initial identification of positive samples, we repeated the preparation of the reaction but excluded coral load and substituted it increasing water in the reaction. We put the sample in the thermocycler with 1 cycle of initial denaturation at 94°C for 2 minutes. The next steps were denaturation with 94°C for 15 seconds, annealing with 56°C for 15 seconds and 52°for 15 seconds and extension for 68°C for 30 seconds. These previous steps were repeated for 40 cycles and a final extension of 1 cycle at 68°C for 1 minute.

We prepared an agarose gel for electrophoresis at 1% to determine the positives. We set a low range DNA ladder of 1.5 kb (Fisher Scientific) in the first well of each line of the agarose gel to determine the approximate size in base pairs of the amplified band. We ran an electrophoresis gel for 40-45 minutes at 100 volts. Samples with coral load are directly put in the electrophoresis gel but for samples without coral load, we added 2 µl of 6X loading dye (Fisher Scientific) to 2.5 µl of PCR product.

We used Haem primer and MalUniv primers and to minimize false negative cases. Haem primers require a nested PCR to function. The set of primers for the inner reaction are HaemNFI (5’-CAT ATA TTA AGA GAA ITA TGG AG-3’) and HaemNR3 (5’-ATA GAA AGA TAA GAA ATA CCA TTC-3’) developed by Fallon (2003). The reaction per sample is composed of 12.5 µl of TopTaq DNA polymerase, 8.5 µl of nuclease free water, 1 µl of HaemNFI as forward primer, 1 µl HaemNR3 as reverse primer and 2 µl of DNA template. As with MalUniv primers, if 2 µl of coral load were added to the reaction and reduced the nuclease free water. We followed the standard protocol for the PCR reaction in the thermocycler established by Fallon (2003).

A second PCR was prepared with the PCR product as template. The second PCR in the nested reaction identified parasites of genus *Plasmodium* and *Haemoproteus*. The reaction was prepared as the first PCR but with the primers HaemF (5’-ATG GTG CTT TCG ATA TAT GCA TG-3’) and HaemR2 (5’-GCA TTA TCT GGA TGT GAT AAT GGT-3’) to detect *Plasmodium* and *Haemoproteus*. The thermocycler protocol for the second PCR reaction is similar to the first PCR reaction but we increased the cycles to 35. We prepared an electrophoresis gel as previously described for MalUniv primers and bands were also detected similarly.

To prepare for sample sequencing, we purified the positive samples using the QIAGEN PCR purification kit protocol. The purpose of sample purification is to clean PCR by-products and increase the probability of obtaining the sample sequence. Purified samples were sequenced using the Sanger Sequencing Service at the Sequencing and Genotyping Facility (SGF) in the University of Puerto Rico, Rio Piedras Campus.

**SI Article. Body condition index**

We describe body condition as the residuals of the relationship:

log(mass) = b_0_ + b_1_ * log(wing-length) + e,

where b_0_ is the intercept, b_1_ the slope, and e ~ N(0,s^2^), where s^2^ represents the variance of the normally distributed residuals. We use a similar equation for the relationship between body mass and tarsus length.
